# Non-Lytic Egression of Infectious Bursal Disease Virus (IBDV) Particles from Infected Cells

**DOI:** 10.1371/journal.pone.0170080

**Published:** 2017-01-17

**Authors:** Fernando Méndez, Nicolás Romero, Liliana L. Cubas, Laura R. Delgui, Dolores Rodríguez, José F. Rodríguez

**Affiliations:** 1 Departamento de Biología Molecular y Celular, Centro Nacional de Biotecnología-CSIC, Madrid, Spain; 2 Instituto de Histología y Embriología de Mendoza - CONICET, Universidad Nacional de Cuyo, Mendoza, Argentina; University of British Columbia, CANADA

## Abstract

Infectious bursal disease virus (IBDV), a member of the *Birnaviridae* family, is responsible for a devastating immunosuppressive disease affecting juvenile domestic chickens. IBDV particles are naked icosahedrons enclosing a bipartite double-stranded RNA genome harboring three open reading frames (ORF). One of these ORFs codes for VP5, a non-structural polypeptide dispensable for virus replication in tissue culture but essential for IBDV pathogenesis. Using two previously described recombinant viruses, whose genomes differ in a single nucleotide, expressing or not the VP5 polypeptide, we have analyzed the role of this polypeptide during the IBDV replication process. Here, we show that VP5 is not involved in house-keeping steps of the virus replication cycle; i.e. genome transcription/replication, protein translation and virus assembly. Although infection with the VP5 expressing and non-expressing viruses rendered similar intracellular infective progeny yields, striking differences were detected on the ability of their progenies to exiting infected cells. Experimental data shows that the bulk of the VP5-expressing virus progeny efficiently egresses infected cells during the early phase of the infection, when viral metabolism is peaking and virus-induced cell death rates are as yet minimal, as determined by qPCR, radioactive protein labeling and quantitative real-time cell death analyses. In contrast, the release of the VP5-deficient virus progeny is significantly abridged and associated to cell death. Taken together, data presented in this report show that IBDV uses a previously undescribed VP5-dependent non-lytic egress mechanism significantly enhancing the virus dissemination speed. Ultrastructural analyses revealed that newly assembled IBDV virions associate to a vesicular network apparently facilitating their trafficking from virus assembly factories to the extracellular milieu, and that this association requires the expression of the VP5 polypeptide.

## Introduction

Viruses are extremophile biological entities enduring rather hostile environments. Indeed, virus success critically depends on the ability to evade aggressions from highly proficient host’s immune systems. As exquisitely exemplified in the poxvirus system, the virus dissemination speed (the lapse between two consecutive cell infection rounds) decisively influences the outcome of the infection [1].

Naked viruses are generally regarded as sluggish lytic pathogens releasing their progeny in a single burst concomitant with host cell disruption [2]. However, research performed during the last two decades has provided a more accurate view, showing that some non-enveloped viruses from different families, e.g. poliovirus (PV, *Enteroviridae*) [3], Hepatitis A (HAV, *Picornaviridae*) [4], bluetongue (BTV, *Reoviridae*) [5] or minute virus of mice (MVM, *Parvoviridae*) [6], are capable of exiting before the onset of cell death.

Infectious bursal disease virus (IBDV), the best characterized member of the *Birnaviridae* family [7], infects juvenile domestic chickens (*Gallus gallus*) causing a severe immunosuppressive disease, known as IBD or Gumboro disease, characterized by the destruction of the Fabricius bursa [8]. Currently prevalent highly pathogenic IBDV isolates, generically known as vvIBDV, decimate chicken flocks causing devastating losses to the poultry industry world-wide. IBDV virions are naked icosahedrons (65–70 nm in diameter, T = 13 symmetry) enclosing a bipartite double-stranded RNA genome [9, 10]. Notwithstanding the conspicuous lack of information concerning virus egress, it has been generally assumed that IBDV behaves as a strict lytic virus.

The IBDV genome has a limited coding capacity, harboring three open reading frames (ORFs) encoding the following proteins: i) VP1, a multifunctional polypeptide with RNA-dependent RNA polymerase activity [11]; ii) a polyprotein that upon successive proteolytic steps releases the two major structural polypeptides (VP2 and VP3) and the virus-encoded protease (VP4); and iii) VP5, a non-structural polypeptide [12–15]. The polyprotein and VP5 ORFs are found at the largest genome segment (segment A). The VP5 coding region, located near the 5’-end of the segment (nucleotides 97–534), partially overlaps the polyprotein ORF (nucleotides 131–3169). According to bioinformatics analyses, the VP5 gene, lacking known viral or cellular orthologs, originated by a gene overprinting mechanism operating on the 5’ end region of an ancestral polyprotein ORF [16]. The presence of the VP5 ORF in viruses belonging to two, i.e. avibirnavirus and aquabirnavirus, out of the four birnavirus genera indicates that this gene originated late during birnavirus evolution.

The VP5 polypeptide has a predicted molecular size of 15-kDa. However, it migrates as a 17-kDa product on SDS-PAGE, thus suggesting that it might be post-translationally modified [15]. Despite the lack of a transmembrane domain, VP5 accumulates at the cytosolic leaflet of different membranous cell compartments, preferentially at the plasma membrane (PM) [17, 18]. This tropism is mediated by the interaction of the VP5 C-terminal polycationic protein region, known as the phosphoinositide (PIP)-binding domain, with integral membrane PIPs [17].

Although VP5 expression is dispensable for virus replication in tissue culture [19], its abrogation causes a dramatic reduction on the virus plaque size [17]. Experimental infections have shown that IBDV VP5 knockout mutants are drastically attenuated, thus evidencing that this protein is a major virulence factor required for both the onset of IBD clinical signs and the development of bursal lesions [19, 20].

Several reports have described the interaction of VP5 with two host cell proteins involved on cell homeostasis, i.e. the regulatory subunit of phosphatidylinositol-4,5-bisphosphate 3-kinase (PI3K) [21], and the mitochondrial voltage-dependent anion channel 2 (VDAC2) protein [22, 23]. The potential involvement of VP5 on apoptosis is under debate with reports supporting both a pro- and anti-apoptotic roles for this protein [20–22, 24]. Indeed, some reports do not associate this polypeptide with the fate of IBDV-infected cells [19, 25].

In spite of its importance, some basic questions, e.g. the temporal regulation of VP5 expression and the influence that this polypeptide might have on specific steps of the IBDV replication program remained unexplored. Here, we describe the results of a systematic analysis aimed to shed light on these important issues.

We have compared the replication of two previously described IBDV viruses, namely WT and VP5-KO, generated by reverse genetics. The genome of the VP5-KO virus harbors a single point mutation that eliminates the VP5 translational initiation site [17]. Results presented here demonstrate that the temporal expression profile of the VP5 polypeptide is akin to other virus-encoded proteins, and that its absence does not significantly affect viral RNA transcription/replication, protein translation, particle assembly or intracellular virus yields. In contrast, we found that VP5 is essential for efficient production of extracellular virus. Transmission electron microscopy (TEM) analyses revealed that a significant fraction of the newly assembled virus particles in cells infected with the WT virus are enclosed within single-membraned vesicles. According to TEM, immunofluorescence microscopy (IF) and virus titration data, these vesicles, exclusively detectable in cells infected with the WT virus, appear to facilitate the release of virus particles from cells harboring an apparently intact PM. Here, we provide first evidence indicating that, as described for other naked viruses, IBDV uses a non-lytic virus egress mechanism that, according to presented experimental data, is dependent upon the expression of the VP5 polypeptide. Our results indicate that the IBDV replication cycle can be divided into two major stages: i) an early phase, when viral RNA transcription/replication, protein translation and virus assembly rates reach their maximum levels. During this phase virus particles are preferentially released using a non-lytic egress mechanism; and ii) a late phase, characterized by a drastic reduction of viral metabolism accompanied by the onset of virus-induced cytopathic effects (CPE) and rapid increase of cell death associated to the release of the remaining intracellular virus progeny.

## Results

### Determination of the temporal expression of the VP5 polypeptide in IBDV-infected cells

Despite the paucity of experimental data, it was suggested that VP5 expression might be restricted to the early stages of the IBDV infection [15]. Indeed, detailed information about the temporal expression is critical for the understanding of the role(s) of this polypeptide. To shed light into this important question, we compared the accumulation kinetics of the VP5 polypeptide with that of the well-characterized VP3 structural protein.

**Fig 1:**
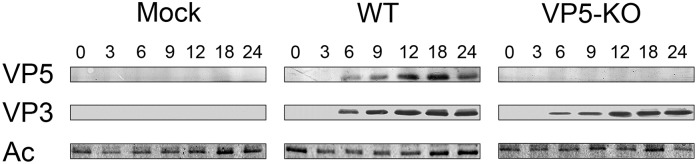
Temporal expression of the VP5 polypeptide. QM7 cell monolayers were mock-infected (Mock) or infected (MOI 3 PFU/cell) with the WT or the VP5-KO virus. Total cell extracts collected at different times PI were subjected to SDS-PAGE followed by Western blot analysis using serum anti-VP5, -VP3 or -Actin (Ac). Numbers above panels indicate the time PI (h) at which samples were harvested.

Preconfluent QM7 cell monolayers were infected with the WT or the VP5-KO virus. Unless otherwise stated, infections described in this report were performed using a multiplicity of infection (MOI) of 3 plaque forming units (PFU) per cell. Mock-infected cultures were used as control in all described experiments. Cultures were harvested at different times, spanning a period covering from 0 to 24 h post-infection (PI). The corresponding extracts were analyzed by Western blotting using either VP5- or VP3-specific antibodies. Protein loading was assessed using anti-actin serum. The experiment was repeated three times with similar results. Fig 1 shows a representative example of gathered data. As expected, an immunoreactive protein band with a migration corresponding to ca. 17-kDa, similar to that described for the VP5 polypeptide, was detected only in extracts from cells infected with the WT virus. The intensity of this band steadily increased with time, showing a behavior akin to that of the VP3 polypeptide, thus evidencing that VP5 expression is maintained throughout the IBDV replication cycle.

### The abrogation of VP5 expression does not affect the IBDV replication program

**Fig 2:**
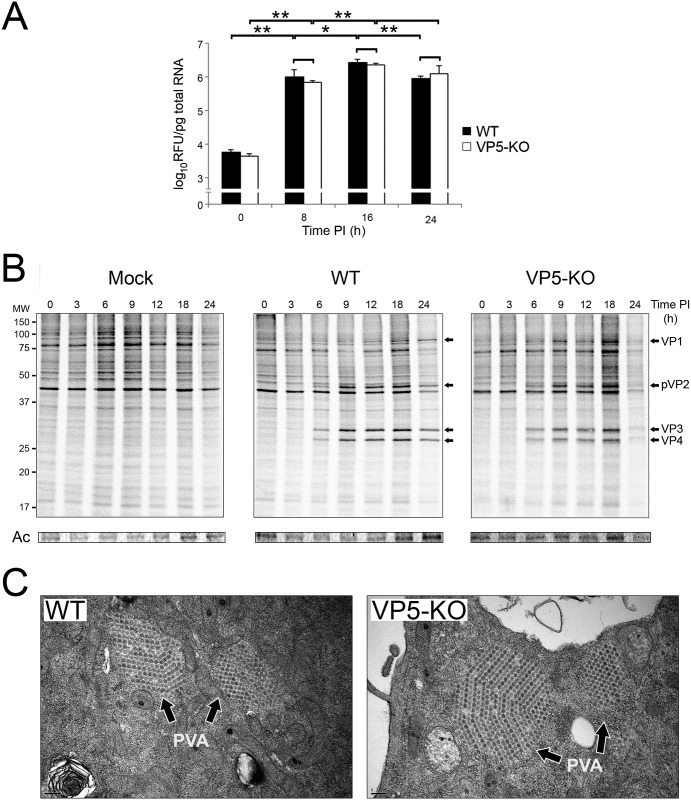
Effect of VP5 abrogation on the IBDV replication program. QM7 cell monolayers were mock-infected (Mock) or infected (MOI of 3 PFU/cell) with the WT or the VP5-KO virus. (A) Accumulation kinetics of IBDV-specific RNAs. Cultures harvested at the indicated times PI were used for the isolation of total RNA. RNA samples were then used for qPCR analysis using primers hybridizing at the VP3 coding region (IBDV genome segment A). Each determination was carried out in triplicate. Presented data correspond to the mean ± the standard deviation of three independent experiments. Black and white bars correspond to data from WT- and VP5-KO-infected cultures, respectively. Brackets indicate pairwise data comparisons. *p<0.01, **p<0.001 as determined by two-tailed unpaired Student’s t-test. (B) Comparative time-course analysis of steady-state protein synthesis. At the indicated times PI, mock-infected (Mock) or infected QM7 cell cultures were metabolically labeled with [^35^S]methionine for 1 h and then harvested. Cell extracts were subjected to SDS-PAGE. After Coomasie-blue staining, gels were dried and used for autoradiography. Upper panels show autoradiograms. The position of molecular mass markers is indicated (MW). Bands corresponding to major IBDV-encoded polypeptides (VP1 to VP4) are pointed by arrows. Panels below autoradiograms correspond to the 42 kDa actin protein (Ac) as detected in the corresponding Coomasie-blue stained gels. (C) Virus assembly. Representative TEM images from samples corresponding to cells infected with the WT or the VP5-KO virus. Samples were harvested at 8 h PI. Paracrystaline virus arrays (PVA) are indicated by arrows. Bars correspond to 0.2 μm.

Although VP5-defective mutant viruses have been previously used in different studies, an assessment of the impact that the abrogation of VP5 expression might have on elemental aspects of the virus replication program, i.e. genome transcription/replication, translation of virus-encoded proteins and virus assembly was as yet lacking. To fill this important knowledge gap, we first analyzed the kinetics of accumulation of IBDV RNA in cells infected with either the WT or the VP5-KO virus. At the indicated times, cultures were harvested and used for the isolation of total RNA. Noteworthy, the integrity of RNAs isolated from cells infected with either virus remained well preserved up to 24 h PI, exhibiting high (˃9) RNA integrity numbers (RIN) [26] similar to those recorded for RNAs from mock-infected cells collected at identical times PI (S1 Fig). Samples were then used for quantitative (q)PCR analysis using primers hybridizing at the VP3 coding region within genome segment A. Data presented in Fig 2A, corresponding to three independent infections, show that the accumulation kinetics of IBDV-specific RNAs were quite similar in both virus infections. Indeed, the statistical analysis of collected data did not reveal significant differences on the accumulation of viral RNA levels amongst both infections at any of the analyzed time points. As evidenced in this figure, RNA transcription/replication is initiated immediately upon infection, reaching a maximal RNA accumulation at 16 h and then slightly decreasing at 24 PI. Noteworthy, the 16–24 PI RNA reduction was statistically significant (p<0.001) only in the case of the WT virus. As expected, similar results were obtained with primers corresponding to the VP1 ORF from segment B (data not shown).

In order to analyze the effect that the absence of the VP5 polypeptide might pose on either the timing or the steady-state synthesis levels of virus-encoded polypeptides, at different times PI cultures were metabolically labeled for 1 h with [^35^S]methionine and then collected for subsequent analysis. Radiolabeled cell extracts were subjected to SDS-PAGE. Gels were stained with Coomasie blue to assess protein loading and, after drying, used for by autoradiography.

Fig 2B shows representative autoradiograms selected from those obtained after three independent experiments. Significant differences were not detected either on the timing or the steady-state levels of protein synthesis in cultures infected with the WT or the VP5-KO mutant. *De novo* synthesis of major IBDV-encoded polypeptides (VP1 to VP4) was first noticeable as early as at 6 h PI. In both cases, maximal steady-state viral protein synthesis levels were maintained from 9 to 18 h and then sharply declined at 24 h PI. Taking into account protein loading data, corresponding to the actin protein bands detected in Coomasie blue-stained gels, we interpret that this reduction reflects both the exhaustion of the translational machinery and the loss of labeled cells due to virus-induced cell death that, as described below, undergoes a rapid increase from 18 to 24 h PI. Noteworthy, the VP5 polypeptide contains a single methionine residue, thus precluding its detection in the autoradiograms.

In order to assess the effect of VP5 on virus assembly, infected cultures were collected at different times PI, i.e. 8, 16 and 24 h, and analyzed by TEM. As shown in Fig 2C, the presence of virus factories containing newly assembled particles forming distinctive honeycomb-like paracrystaline virus arrays (PVA) was already evident in both infections at 8 h PI. Indeed, the identity of PVAs was confirmed by immuno-EM using anti-VP2 serum (S2 Fig). Although, as shown below, both the relative abundance and PVA average size increased at later times PI, differences between WT- and VP5-KO-infected cells were not detected. Indeed, these results indicate that the absence of the VP5 polypeptide does not affect particle assembly.

### Effect of VP5 expression on virus progeny yields

We have previously shown that the abrogation of VP5 expression significantly reduces virus progeny yields [17]. In order to get a deeper insight about the contribution of the VP5 protein to the replication process, it was of critical importance to get a detailed comparative assessment of the one-step intra- and extracellular growth curves of the WT and the VP5-KO viruses. These experiments were performed by infecting preconfluent QM7 monolayers. Presented data correspond to four independent experiments.

**Fig 3:**
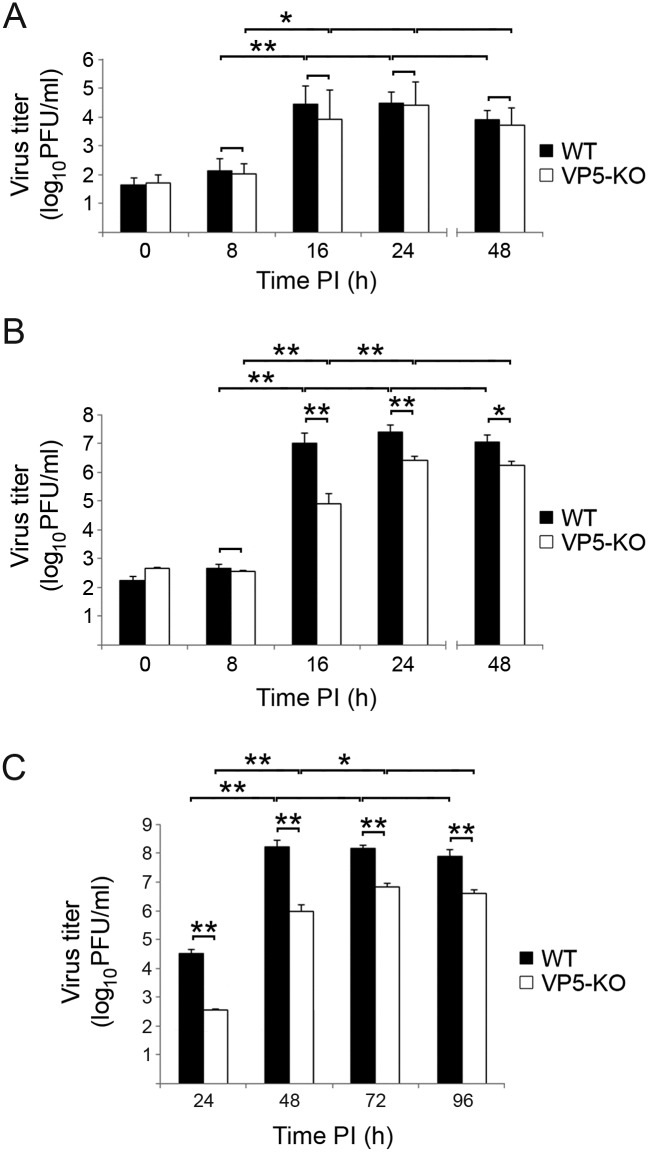
Effect of VP5 expression on virus progeny yields. Comparative time-course analysis of virus progeny yields in QM7 cell cultures infected at high MOI (3 PFU/cell) with the WT or the VP5-KO virus. (A) Intracellular virus titers. (B) Extracellular virus titers. (C) Comparative time-course analysis of total virus progeny yields in QM7 cell cultures infected at low MOI (0.05 PFU/cell) with the WT or the VP5-KO virus. Black and white bars correspond to cultures infected with the WT and the VP5-KO virus, respectively. Presented data correspond to the mean ± the standard deviation of three independent experiments. Virus titrations were carried out in triplicates. Brackets indicate pairwise data comparisons. *p<0.01, **p<0.001 as determined by two-tailed unpaired Student’s t-test.

As shown in Fig 3A, the kinetics of intracellular virus accumulation was almost identical in both infections, reaching a plateau at 16 h PI. Statistically significant differences were not detected when comparing intracellular virus titers produced upon infection with either virus, thus arguing against a possible role of the VP5 polypeptide during the packaging of the viral genome.

In contrast, comparison of extracellular virus titres (Fig 3B) evidenced the existence of significant differences on the kinetics of extracellular virus production. Whilst extracellular WT titers reached a plateau at 16 h PI, highest extracellular VP5-KO titers were attained at 24 h and then declined at 48 h PI. Moreover, from 16 h PI onwards, extracellular virus titers recorded in cultures infected with the WT virus were significantly higher (>2 log_10_ units at 16 h PI, and ca. 1 log_10_ units at 24 and 48 h PI) than those found in VP5-KO-infected cultures.

This series of experiments suggested that abrogation of VP5 expression specifically impairs the ability of the virus progeny to exiting infected cells. In order to further assess this hypothesis a comparison of the growth kinetics of both viruses in cultures infected at low MOI (0.05 PFU/cell) was performed. As shown in Fig 3C, both viruses showed substantially different growth kinetics. Whilst maximal virus titers in cultures infected with the WT virus were detected at 48 h PI, VP5-KO highest titers were scored at 72 h PI. Additionally, as expected from data presented above, infection with the WT virus results in the production of significantly higher virus yields (over 100-fold at 24 and 48 h PI and ca. 10-fold at 72 and 96 h PI, respectively) than those recorded in VP5-KO-infected cultures.

### Effect of VP5 on cell death kinetics

We have previously described that overexpression of the VP5 polypeptide using a vaccinia virus inducible expression vector causes a major deregulation of cell homeostasis, eventually leading to cell disruption [27]. Although the VP5 expression levels in IBDV-infected cells are much lower than those observed using the described vaccinia virus vector, it seemed feasible that, the accumulation of this polypeptide might promote cell lysis, thus facilitating the release of the virus progeny. To explore this hypothesis we performed a series of real-time assays to comparing cell death kinetics from 0 to 48 h PI in cultures infected with the WT and the VP5-KO viruses.

After infection, monolayers were washed and incubated in fresh medium supplemented with a cell-impermeant fluorescent dye, which upon PM destabilization penetrates the cell. The subsequent binding of the dye to cellular DNA allows identifying damaged cells. Cultures were monitored using IncuCyte ZOOM live cell imaging system.

**Fig 4:**
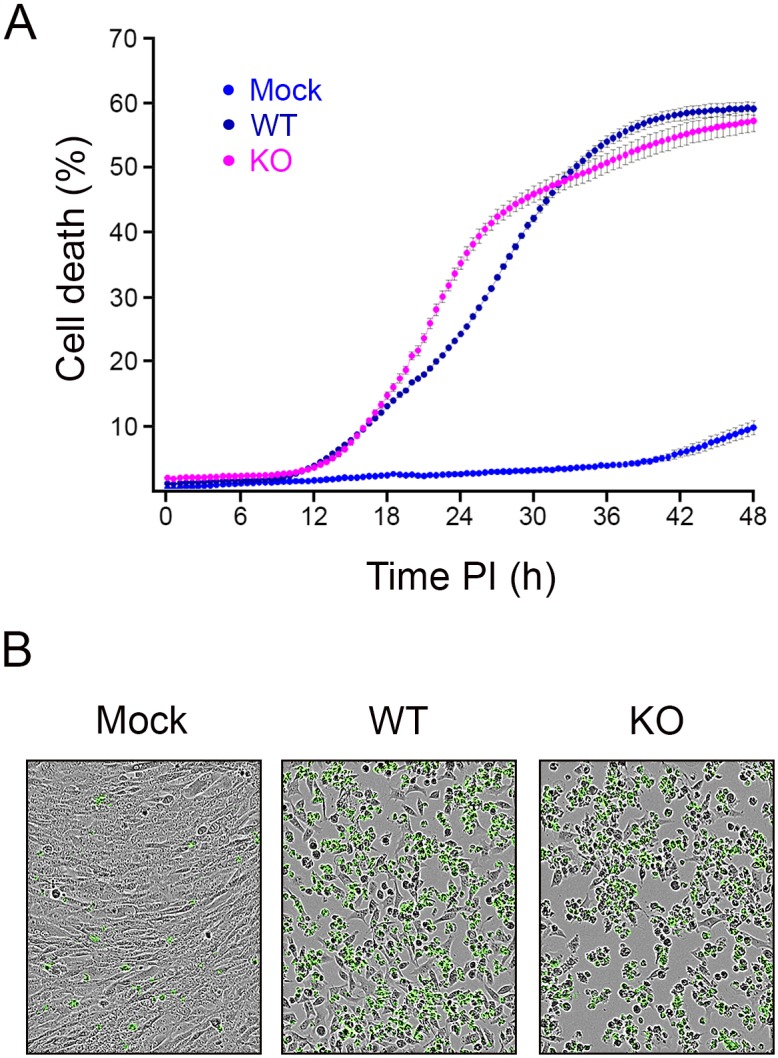
Quantitative real-time cell death analysis. Preconfluent QM7 cell monolayers were mock-infected (Mock) or infected (MOI of 3 PFU/cell) with the WT or the VP5-KO virus. Infected cultures were maintained for 48 h in medium supplemented with a fluorescent dye exclusively staining cells with damaged PM. (A) Cell death quantification. Cultures were monitored every 30 min using an IncuCyte ZOOM live cell imaging system. Four randomly selected monolayer locations were imaged in each well over the entire time course. The IncuCyte Zoom software was used to automatically score and quantify cell death. Presented data corresponds to three independent infections involving the quantification of data from a total of 12 video recordings per sample. (B) Cell death imaging. Representative images from the different cultures captured at 48 h PI.

As shown in Fig 4A, death rates recorded in cultures infected with the WT and the VP5-KO virus were undistinguishable up to 18 h PI, reaching in both cases ca. 10% of the infected cells. From 18 to 30 PI the death rate in cultures infected with the VP5-KO mutant was slightly, yet significantly (p<0.01), higher than that recorded in WT-infected in cultures. Interestingly, after this period death rates were quite similar in both infections. As expected, the death rate in mock-infected cultures was much lower, reaching a 10% at 48 h. Representative images from mock- and virus-infected cells, corresponding to 48 h PI, are presented in Fig 4B. Illustrative video recordings of these experiments are as provided as supplemental information (S1 to S3 Videos).

Results from these experiments rule out a possible role of VP5 in promoting cell lysis early during infection. Our data conclusively shows that at 16 h PI, the time point when maximal WT intra- and extracellular virus titers are reached (Fig 3), only 12% of the total cell population exhibited a leaky PM. Indeed, taking together virus titration and cell death kinetics data, we conclude that, in contrast to what happens in cultures infected with the VP5-deficient virus, the bulk of the WT extracellular progeny is released early during the infection (from 8 to 16 h PI) from cells harboring undamaged PMs.

### Effect of VP5 expression on virus dissemination

Single-step virus growth data described above showed the presence of high extracellular virus titers as early as at 16 h PI in cells infected with the WT virus. This observation prompted us to assess virus dissemination at single cell level. To this end, QM7 cell monolayers were infected at low MOI (0.005 PFU/cell) with the WT or the VP5-KO virus. After virus adsorption, cultures were washed with fresh medium, and then covered with semi-solid medium to prevent long-range diffusion of virus particles. At different times PI, i.e. 8, 16, or 24 h, the semi-solid medium was lifted. Cell monolayers were immediately fixed and processed for immunofluorescence using an antibody specific for the IBDV VP2 capsid polypeptide, followed by DAPI staining. Samples were visualized by fluorescence microscopy.

**Fig 5:**
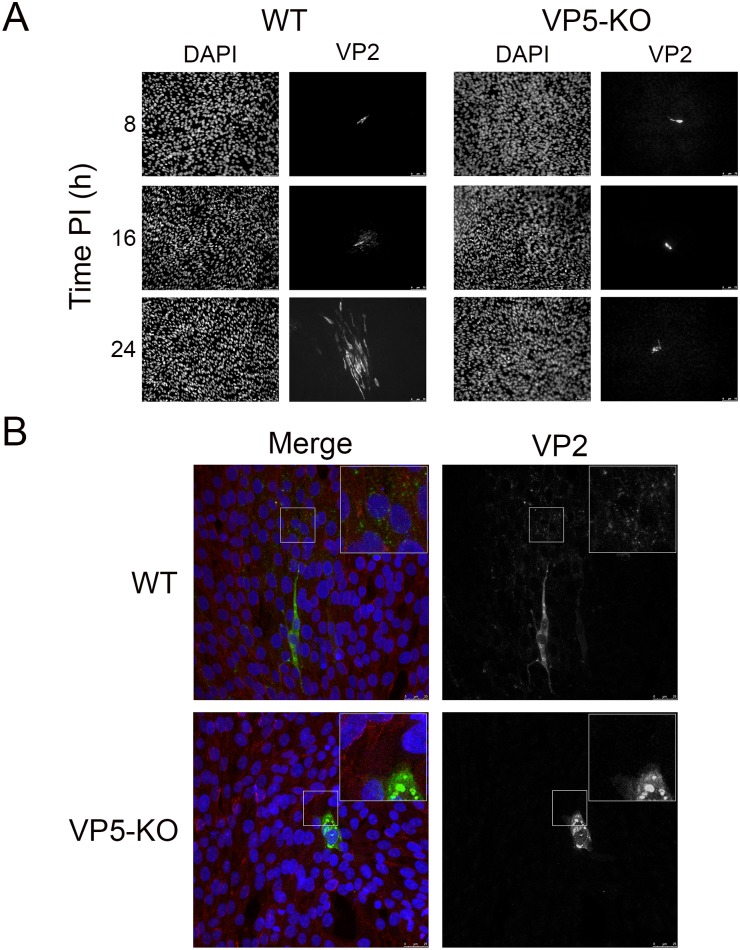
Effect of VP5 expression on IBDV dissemination. QM7 cell monolayers were infected at low MOI (0.005 PFU/cell) with the WT or the VP5-KO virus. After virus adsorption, cultures were washed twice with fresh medium to remove unbound virions, and covered with semi-solid medium. (A) Single-cell virus spreading. At the indicated times PI, the semi-solid medium was lifted and monolayers processed for immunostaining with anti-VP2 serum (VP2). Nuclei were stained with DAPI. Samples were then visualized by fluorescence microscopy. Fluorescence signals were recorded separately by using appropriate filters. Presented micrographs are characteristic examples of images recorded at the indicated times PI. Left- and right-hand panels show the DAPI and VP2 staining of the same culture areas, respectively. Bars correspond to 75 μm. (B) Visualization of virus release at single-cell level. Samples collected at 16 h PI were immunostained with anti-VP2 serum (green) and -Pan cadherin (red). Cell nuclei were stained with DAPI (blue). Samples were analyzed using confocal laser scanning microscopy. Left-hand panels show the overlay of the three fluorescent signals (Merge). Right-hand panels show the VP2 signal. Insets show a higher magnification (x2.5) of boxed areas. Bars correspond to 25 μm.

The results of this study are summarized in Fig 5A, displaying a representative set of images corresponding to both infections. First signs of infection were detected at 8 h PI. As expected, at this time point, less than 0.01% of the total cell population was positive for VP2 staining. VP2-positive cells were completely surrounded by uninfected (VP2-negative) cells. The overall situation was maintained at 16 h PI. Interestingly, at this time point, the surroundings of over 50% of the VP2-positive cells in WT-infected cultures were sprinkled with bright VP2-specific puncta. This particular staining pattern was absent in cells infected with the VP5-KO mutant. A rather conspicuous difference between these two infections was detected in samples collected at 24 h PI. Whilst the VP2 signal in VP5-KO-infected cultures was as yet restricted to single cells, monolayers infected with WT virus were embossed with discrete groupings encompassing ca. 25–50 VP2-positive cells.

Images recorded at 16 h PI suggested the possibility of detecting newly egressed particles from WT-infected cells. To further explore this possibility, samples collected at 16 h PI were processed as described above. In this case, to visualize cell boundaries, in addition to the anti-VP2 serum, samples were incubated with a mouse monoclonal antibody against cadherins, a group of cellular transmembrane proteins. Samples were visualized using confocal laser scanning microscopy (CLSM). The results of this analysis are summarized in Fig 5B which shows representative images corresponding to cultures infected with the WT or the VP5-KO virus. As expected, cultures infected with the WT virus contained single VP2-positive cells surrounded by VP2-negative cells. Infected cells exhibited a flat, elongated, healthy appearance containing one or two seemingly intact nuclei. The intracellular VP2 signal was formed by several large ring-like accretions as well as disseminated puncta. Remarkably, large areas surrounding a significant fraction (˃50%) of the VP2-positive cells were spotted with a myriad of VP2-fluorescent puncta. This staining pattern, that in our interpretation reflects the presence of newly egressed virus particles, was not found in samples from cultures infected with the VP5-KO mutant.

In good agreement with virus titration and real-time cell death data, presented IF results indicate that a significant fraction of the WT virus progeny egresses infected cells before the onset of cell death, and that this ability is lost in the VP5-KO virus.

### Electron microscopy analysis of cells infected with WT and VP5-KO viruses

Data described above suggested the interest of comparing the ultrastructure of WT- and VP5-KO-infected cells. Preconfluent QM7 cell monolayers were grown on sapphire disks, and then infected with the WT or VP5-KO viruses. Samples were collected 16 and 24 h PI, and processed for TEM analysis.

**Fig 6:**
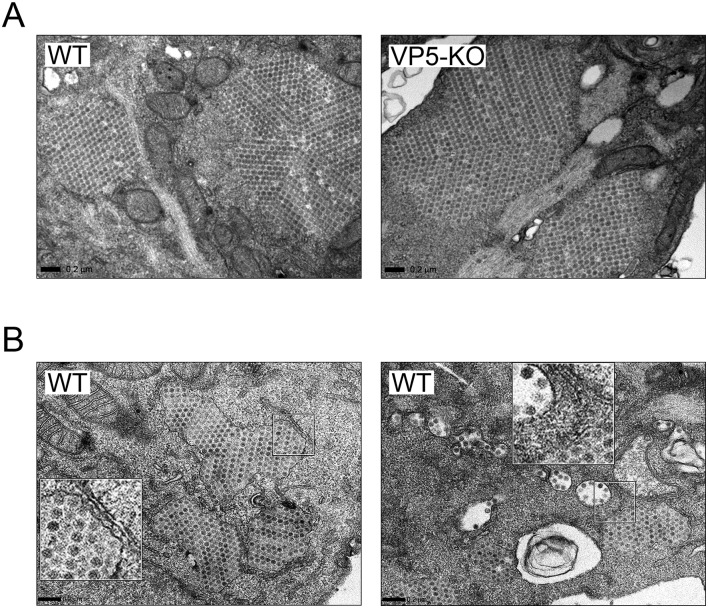
Ultrastructural analysis of IBDV-infected cells. QM7 cell monolayers infected (3 PFU/cell) with the WT or the VP5-KO virus were collected at 16 h PI and processed with TEM analysis. (A) PVA assembly. Panels show characteristic examples of PVAs detected in the cytoplasm of cells infected with either virus. (B) Detection of PVAs wrapped up by single membranes and VCVs. Representative images showing details of the cytoplasm of WT-infected cells containing several PVAs surrounded by a single membrane (left panel) and three membrane-enclosed PVAs along with VCVs (right panel). Insets show a higher magnification (x2.5) of boxed areas. Bars correspond to 0.2 μm.

**Fig 7:**
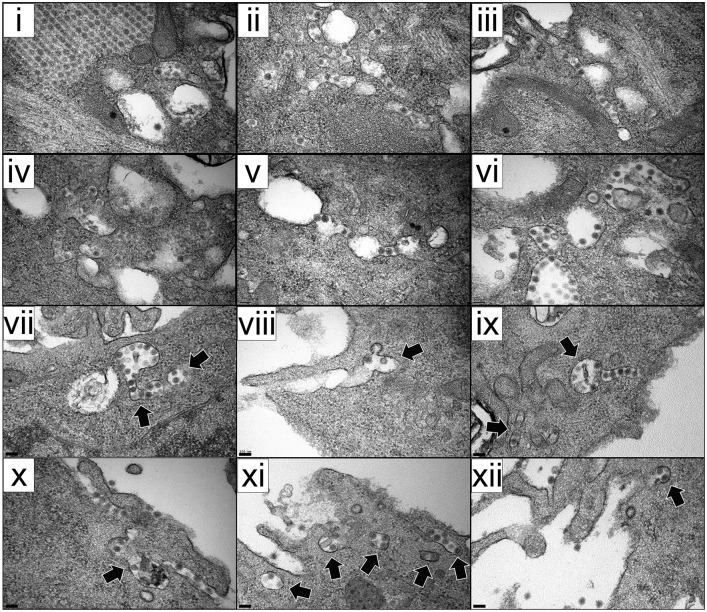
VCV gallery. Micrographs showing VCVs detected in QM7 cells infected (3 PFU/cell) with the WT virus. Samples were prepared at 16 h PI. Some VCVs located either near or apparently fused to the PM are indicated by arrows. Bars correspond to 0.1 μm.

**Fig 8:**
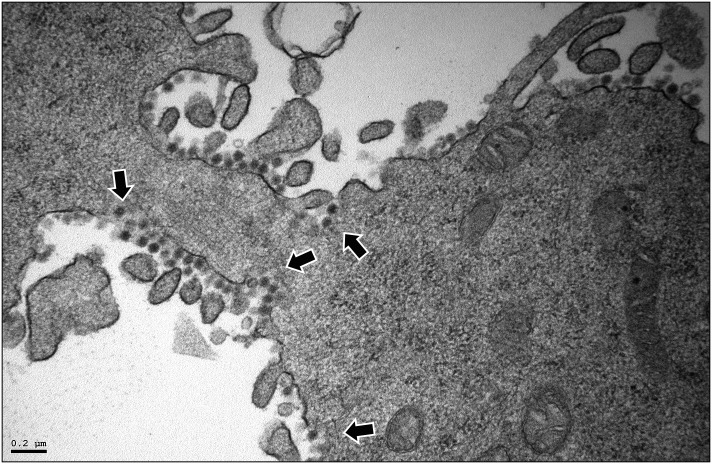
Newly released IBDV virions. Micrograph showing a detail a WT-infected (3 PFU/cell) QM7 cell containing IBDV virions bound to the external PM surface. The sample was collected and processed for TEM at 16 h PI. Arrows point PM perturbations likely associated to virus particle release. Bar indicates 0.2 μm.

Taking into account data described above, our analysis was mainly focused on samples collected at 16 h PI. At this time PI, the cytoplasm of cells infected with either virus was studded with distinctive PVAs, harboring large numbers of tightly packed virus particles, in most cases located in close proximity to mitochondria (Fig 6A). Noteworthy, a large fraction of the PVAs detected in cultures infected with the WT virus was either partially or completely surrounded by single membranes (Fig 6B). Additionally, along with PVAs, the cytoplasm of cells infected with the WT virus contained abundant “free” (as opposed to tightly packed virions found in PVAs) virus particles exhibiting both a polygonal contour and a diameter identical to that of PVA-associated virions (Fig 6B, right panel). These “free” virus particles, either alone or in small, loose groupings, were invariably enclosed within pleomorphic, single-membrane, virus-containing vesicles (VCV) scattered throughout the cytoplasm, in many instances close to the PM. A gallery of representative VCV images is presented in Fig 7. The external PM surface of a large fraction (>50%) of the WT-infected cells (PVA-containing cells) was profusely decorated with virus particles (Fig 8). Regrettably, stand-still TEM images do not allow ascertaining whether particles, as those shown in Fig 8, are exiting or entering cells. Indeed, virus titration and dissemination data (Figs 3 and 5) strongly back the first option. Interestingly, the continuity of the PM within the vicinity of seemingly egressing particles is either disturbed or lost (Fig 7 panels viii-xii, and Fig 8), suggesting that virus release might be the result of a VCV-PM fusion process. Certainly, this hypothesis should be carefully evaluated using more sophisticated imaging approaches and specific pharmacological treatments.

In spite of an intensive search, PVA-wrapping membranes, VCVs or cell-surface attached virus particles were not found in samples from cultures infected with the VP5-KO virus.

Inspection of samples infected for 24 h exhibited similar, yet exacerbated, infection signs to those observed at 16 h PI, i.e. the presence of large VPAs, accompanied in WT-infected cells by VCVs and abundant extracellular membrane-bound virions (data not shown). Additionally, these TEM inclusions contained cellular debris along with PVAs, VCVs (only present in samples from WT-infected cells), and tubular assemblages akin to those described for type II VP4 tubules [28], probably corresponding to cells lysed during sample preparation (S3 Fig).

## Discussion

### Characterization of VP5 expression, and its role on the IBDV replication cycle

Experimental data showing the expression of the VP5 polypeptide in IBDV-infected cells were first described by Mundt et al. [19]. Based on results from a single immunoprecipitation assay using extracts from infected cells collected from 1 to 6 h PI, authors concluded that the temporal regulation of VP5 expression might differ from that of other IBDV-encoded polypeptides, being shut-off early during the IBDV replication cycle. No further reports specifically dealing with the temporal control of VP5 expression have been published to date. Our data unambiguously shows that the intracellular amount of VP5 steadily increases with time, closely paralleling the accumulation kinetics of the structural VP3 polypeptide. Indeed, these results rule out the initial suggestion that the translation of the VP5 ORF might be selectively arrested before the completion of the IBDV replication cycle, and thus argue against the possibility that this polypeptide might exert a transient role during the initial stages of the infection.

Despite efforts from different laboratories, including ours, to establishing both the specific function(s) and the role of the VP5 protein on IBDV pathogenesis, there was a conspicuous lack of experimental data concerning its possible contribution to elemental steps of the IBDV replication program. Our data conclusively show that abrogation of the VP5 ORF does not affect “house-keeping” functions such as genome transcription/replication, mRNA translation or particle assembly. In agreement with these findings, we have determined that VP5 expression is irrelevant for intracellular virus production.

Maximal intra- and extracellular WT virus yields are reached during the 8–16 PI period. This period concurs with that of highest viral metabolic activity and, according to real-time cell death analyses, with minimal virus-induced cell death rates (ca. 6%). A key conclusion drawn from our experiments is that a substantial fraction of the WT virus progeny is released from metabolically-active cells. The landscape is quite different in cultures infected with the VP5-deficient virus. Although both viral metabolism and intracellular virus titers follow kinetics akin to those of recorded in WT-infected cells, maximal extracellular virus titers production is significantly delayed, being reached between 16–24 h PI, coinciding in time with the burst of virus-induced cell death. Certainly, our data clearly evidences that VP5 is a key element controlling the release of the infectious progeny, and consequently virus dissemination.

Presented data indicate that, similarly to other naked viruses, IBDV utilizes two independent releasing mechanisms. The first one, dependent upon VP5 expression, responsible for the egress of infectious particles from live, metabolically active, cells; and a second one, associated to cell lysis, facilitating the release of the remaining progeny together with the intracellular content. Critical cellular decisions involving complex dynamic signaling pathways, such as virus-induced cell death, are highly dependent upon cell-to-cell variability [29]. Consequently, some temporal overlapping of both mechanisms is only to be expected when analyzing virus release at population level.

It is worth noting that extracellular virus yields are consistently higher than intracellular ones. This difference is particularly remarkable, over 100-fold, in cultures infected with the WT virus. Although we have not investigated the cause(s) underlying this finding, we envisage two possible explanations for these differences. It seems feasible that a fraction of the intracellular particles might correspond to immature particles incapable of initiating a productive infection. An alternative explanation is related to assembly of large PVAs in IBDV-infected cells which are rather abundant in samples used for intracellular virus titrations. PVAs are highly ordered and stable superstructures, in some cases harboring thousands of particles. The intrinsic stability of this superstructure might largely reduce the infective vs. physical particle ratio in samples used for intracellular virus titrations. Although the molecular mechanism underlying the PVA assembly and stability has been described [30], its biological significance remains unexplored. It is tempting though to speculate that PVAs might play a role on the horizontal transmission of the virus, akin to that of well characterized polyhedra assembled in cells infected by members of the *Baculoviridae* family [31]. This hypothesis would beautifully fit with a preferential role of the newly described non-lytic virus egression on virus spreading at individual level, whilst lytic egression, releasing environmentally stable PVAs, might serve to ensuring long-term individual-to-individual IBDV transmission.

### Non-lytic IBDV egress

Formal demonstration at single cell level that a non-enveloped virus uses a non-lytic egress pathway is a difficult matter. It requires solid proof that virus spreading is not the result of a small number of lysed cells in cultures under study. This challenge has been elegantly overcome by Bird and cols. using a real-time, single-cell assay allowing a precise scrutiny of the poliovirus dissemination process [3]. This assay was based on the use of a recombinant poliovirus constitutively expressing the green fluorescent protein. Unfortunately, our attempts to generating recombinant IBDV viruses expressing heterologous marker genes have repeatedly failed. In view of this experimental limitation, we resorted to a classical immunostaining approach. Data presented here confirm that under stringent experimental conditions, the spreading of the WT virus is much faster than that of its VP5-KO counterpart. Using this approach we also observed that, early during infection (16 h PI), a significant number of cells infected with the WT virus were surrounded by VP2-specific puncta that we interpret as newly egressed virus particles. Seemingly virus-releasing cells had a healthy aspect, strongly suggesting that surrounding virus particles were not the product of cell lysis. In agreement with virus titration and dissemination data, healthy looking virus-releasing cells were exclusively detected in cultures infected with the WT virus.

Our ultrastructural analysis revealed two previously undescribed structures, likely sharing a common origin, and exclusively detectable within the cytoplasm of WT-infected cells: i) single membranes wrapping up PVAs; and ii) a complex network, formed by single-membrane VCVs, enclosing large numbers of individual virus particles. We have also detected the presence of VCVs in DF-1 (spontaneously transformed chicken embryo fibroblasts) infected with the WT virus (data not shown), thus indicating that this is common structure in IBDV-infected cells.

TEM images suggest that VCVs might be interconnected with PVAs, and that IBDV particles traffic along this vesicular network extending across the cytoplasm of infected cells. TEM images also suggest that VCVs might act as exocytic vesicles fusing with the PM, and thus facilitating the release of its infectious cargo to the extracellular space.

Indeed, our results raise major biological questions concerning both the origin of membranes wrapping up both PVAs and “free” virus particles and the molecular mechanism underlying the role of the VP5 polypeptide on the biogenesis or recruitment of this membrane system. Hopefully, ongoing tomographic analyses and pharmacological treatments will provide a better understanding about these newly described structures.

In a recent review article [32], Bird and Kirkegaard proposed three alternative mechanisms capable of sustaining non-lytic egression of naked viruses. Our TEM data clearly argues against first proposed mechanism, the blebbing of virus particles from the PM of infected cells, for the release of IBDV virions. The two remaining alternatives are based on the exploitation of major cellular recycling routes, i.e. the multivesicular body (MVB) pathway and the autophagy pathway, encompassing the biogenesis of exocytic vesicles allowing the delivery of cytoplasmic content to the extracellular milieu from intact cells. As described above, we have recently shown that VP5 specifically binds different PIP molecules, exhibiting a clear preference for monophosphate PIP species, including PtdIns3P [17]. Additionally, it has been reported that VP5 interacts with the regulatory p85α subunit of the PI3K (phosphatidylinositol 3-kinase) [21]. These previous findings suggest that VP5 might interfere PIP-mediated signaling, in particular with that mediated by PtdIns3P, a key molecule in the regulation of both the MVB and the autophagy pathways [33]. We have shown that the IBDV replication process occurs in direct association with endosomal membrane compartments [34]. This observation evokes the possibility that IBDV might hijack the MVB pathway for the exportation of infectious particles. However, at this point it is difficult to envisage a process allowing the incorporation of naked IBDV particles into the lumen of endocytic vesicles. Concerning the autophagy pathway, it has been recently published that the interaction of IBDV particles with one of the described IBDV virus receptors, HSP90AA1 (heat shock protein 90 kDa α class A member 1), causes the inactivation of the AKT-mTOR pathway, triggering a transient autophagic response in infected cells [35]. This observation suggests the possibility that IBDV might subvert the autophagy pathway in order to promote virus release. Indeed, research to gain a deeper insight about mechanism(s) sustaining non-lytic IBDV egress is ongoing.

Although our understanding of the underlying molecular events is as yet remote, we provide solid evidence that IBDV uses a VP5-dependent mechanism sustaining the non-lytic release of infectious particles early during its replication cycle, hence opening new venues to exploring the molecular mechanisms underlying IBDV virulence, and for the development of new strategies to control IBD.

As described above, the incorporation of the VP5 ORF to the birnavirus genetic repertoire was, in evolutionary terms, a late event [16]. It seems likely that the advent of this new polypeptide, sustaining an efficient cell exiting mechanism, might have significantly modified virus-host interactions and, probably, the virus lifestyle. Some birnavirus species, including those from the blosna- and entomobirnavirus genera, do not harbor this genetic element. A comparative analysis of birnavirus egression mechanisms and their relationship to virus pathogenesis might shed light into the evolution of this intriguing virus family [36].

## Materials and Methods

### Cells, viruses, infections

QM7 (quail muscle myoblasts, ATCC number CRL-1962) were grown in Dulbecco’s modified Eagle's medium (DMEM) supplemented with penicillin (100 U/ml), streptomycin (100 mg/ml) and 5% fetal calf serum (FCS) (Sigma). The WT and VP5-KO IBDV viruses used in this report are derivatives of the IBDV Soroa strain, a cell-adapted, serotype I virus, generated by reverse genetics in our laboratory [17]. Viruses were propagated as described [10]. Infections were performed by diluting virus stocks in DMEM. After washing with PBS, monolayers were incubated with virus suspensions at 37°C for 1 h. After virus adsorption, the medium was replaced with fresh DMEM supplemented with 2% FCS. Virus titrations were performed in triplicate using an immunostaining-based plaque assay as previously described [17]. For virus titrations, cultures were collected by gentle cell scrapping. Cell suspensions were subjected to low speed centrifugation (2,000xg for 5 min) to remove cell debris. The resulting supernatants were used to determine extracellular virus titers. Cell pellets were suspended in fresh medium and subjected to three sonication cycles of 10 sec at 4°C using water cup bath Branson Sonifier 450 (Danbury, CT), to dislodging virus particles from cell debris and then used to determine intracellular virus titers.

### Quantitative PCR analysis

Total RNA from mock-infected or infected cell cultures was isolated using the RNeasy mini kit (Qiagen). Isolated RNAs were reverse transcribed into complementary DNA (cDNA), using random-primer, 500 ng of RNA and Super Script III (Invitrogen) according to the manufacturer’s protocol. The cDNA was then subjected to qPCR using two specific primers, i.e. 5’-AAGGGCAGCTACGTCGATCTAC and 5’-TGGCAACTTCGTCTATGAAAGC, hybridizing at the VP3 coding region of IBDV segment A. qPCR reactions were performed in triplicate using Power SYBR Green PCR Master Mix (ThermoFisher Scientific) and an Applied Biosystem 7500 Real-Time PCR System instrument. Reactions were performed as follow: 2 min at 50°C, 10 min at 95°Cfollowed by 40 cycles of 15 s at 95°C, 1 min at 60°C, finally by 15 s at 95°C, 1 min at 60°C, 30 s at 95°C and 15 s at 60°C to build the melt curve. IBDV RNA loads were calculated from threshold cycle (Ct) values by linear regression using the standard curve.

### SDS-PAGE, western blot and autoradiography

Samples used for Western blot analysis were prepared by removing media from cell monolayers and suspending cells in iced-chilled disruption buffer (0.5% Triton X-100; 50 mM KCl; 50 mM NaCl; 20 mM Tris-HCl pH 7.5; 1 mM EDTA, 10% glycerol) supplemented with protease inhibitors (Complete mini EDTA free protease inhibitor cocktail; Roche). Cell lysates were mixed (v/v) with 2x Laemmli’s sample buffer (125 mM Tris-HCl pH 6.8; 4% SDS; 0. 5% bromophenol blue; 10% glycerol; and 10% β-mercaptoethanol). Electrophoreses were performed in 12% SDS-PAGE, followed by electroblotting onto nitrocellulose membranes. Immunoblots were incubated with blocking buffer (PBS containing 5% non-fat dry milk) for 1 h at 22°C, washed with PBS, and then incubated at 4°C overnight with the corresponding primary antibodies diluted in blocking buffer. Antibodies used in this study were rabbit polyclonal sera specific for IBDV VP2 [37], VP5 [17] and a mouse monoclonal specific for β-actin (Sigma). After incubation with primary antibodies, membranes were incubated with either goat anti-rabbit IgG-Peroxidase conjugate (Sigma) or goat anti-mouse IgG-Peroxidase conjugate (Sigma). Immunoreactive bands were detected by enhanced chemiluminescence reaction (GE Healthcare).

For metabolic labeling, cell monolayers were washed twice with methionine-free DMEM. Thereafter, cultures were incubated for 1 h with 100 μCi/ml of [^35^S] methionine, washed twice with PBS, and suspended in Laemmli’s sample buffer. Protein samples were subjected to 12% SDS-PAGE, and the polyacrylamide gels were fixed, stained with Coomasie-blue to visualize protein bands, and dried. Radioactive signals were detected with a Storm gel imaging system (Molecular Dynamics).

### Quantitative real-time cell death assays

Preconfluent cell monolayers grown in 24 well plates were infected with the WT or the VP5-KO virus. After virus adsorption, monolayers were gently washed and covered with fresh medium supplemented with 2% FCS and IncuCyte Cytotox Green reagent (Essen Bioscience, Inc., Ann Arbor, MI) following manufacturer’s instructions, and placed within an IncuCyte ZOOM System apparatus. Cultures were monitored every 30 min using a 10x objective lens. Four randomly selected monolayer locations were imaged in each well over the entire time course. IncuCyte Zoom software was used to automatically score and quantify cell death. Presented data corresponds to three independent infections involving the quantification of a total of 12 video recordings.

### Immunofluorescence analysis

Cells seeded onto μ-24 well plates (Ibidi) were infected with indicated viruses. After infection, monolayers were washed twice with PBS, covered with semisolid medium (DMEM, 0.7% nobel agar, 2% FCS) and maintained under normal culture conditions. At the indicated times PI, the semisolid medium was carefully removed, monolayers washed twice with PBS, fixed with methanol/acetone (v/v) for 5 min at -20°C, and then air-dried. Fixed monolayers were blocked for 20 min using a solution of PBS containing 5% FCS, and incubated with rabbit anti-VP2 for 1 h. After extensive washing with PBS, monolayers were incubated with a mouse monoclonal against cadherin (anti-Pan cadherin; Sigma) for 1 h. Thereafter, samples were incubated with goat anti-rabbit IgG coupled to Alexa 488 (green) and goat anti-mouse IgG coupled to Alexa 594 (red). Cell nuclei were stained with 2-(4-amidinophenyl)-1H-indole-6-carboxamidine (DAPI; Sigma) diluted in PBS for 30 min. All incubations were performed at room temperature (RT). Finally, wells were covered with PBS. Samples were visualized by epifluorescence using a Leica TCS-Sp5 microscope confocal system. Fluorescent signals detected by CLSM were recorded separately by using appropriate filters. Images were captured using the LAS-AF v.2.6.0 software package (Leica Microsystems).

### Transmission electron microscopy

QM7 cells grown on 3 mm sapphire disks (Engineering Office M. Wohlwend GmbH. Switzerland) were mock-infected or infected with the WT or the VP5-KO virus. At indicated times PI, cultures were maintained for 1 h at RT in fixing solution (HEPES 0.4 M pH 7.2; 2% glutaraldehyde; 1% tannic acid). Samples were then washed with HEPES 0.4 M pH 7.2 and incubated for 45 min at 4°C in post-fixing solution (1% osmium tetroxide and 0.8% potassium ferricyanide in distilled water), and then processed for embedding in EMbed 812 resin (Electron Microscopy Sciences) following the manufacturer’s instructions. Ultra-thin sections of the samples were stained with saturated uranyl acetate and lead citrate. Micrographs were recorded with a Jeol 1200 EXII electron microscope operating at 100 kV.

### Statistics

Graphpad Prism version 5.03 software (GraphPad Software, La Jolla, CA) was used to determine statistical significance using Student unpaired two-tailed t test. p value cutoffs and notation were used as follows: *p<0.05, and **p<0.01.

## Supporting Information

S1 Fig Total RNAs isolated from infected QM7 cells. QM7 cell monolayers were mock-infected (Mock) or infected (MOI of 3 PFU/cell) with the WT or the VP5-KO virus. At the indicated times PI, cultures were harvested and processed for the isolation of total RNA using the RNeasy kit (Qiagen). Isolated RNAs were subjected to Tris-borate-EDTA 1% agarose gel electrophoresis. The gel was stained with SYBR Safe (ThermoFisher Scientific). The positions of the 28S and 18S ribosomal RNAs are indicated.
(TIF)

S2 Fig Identification of IBDV particles by immunoelectron microscopy. QM7 cells were infected (3 PFU/cell) with WT IBDV. At 16 h PI monolayers were fixed in situ with 4% paraformaldehyde 0.1% glutaraldehyde in PBS at 4°C for 4 h. After fixation, cell pellets were suspended in glycerol and frozen in liquid ethane. Frozen specimens were transferred to a Riechert-Jung AFS freeze-substitution unit (Leika) and maintained at 90°C in a mixture of methanol and 0.5% (W/V) uranyl acetate for 48 h. Thereafter, samples were infiltrated in Lowicryl KYM (EML laboratories) at 30°C. Polymerization was induced with UV light. Ultrathin sections of the samples were immunolabeled with anti-VP2 serum followed by incubation with goat anti-rabbit IgG conjugated to 5-nm colloidal gold. Micrographs were recorded with a Jeol 1200 EXII electron microscope operating at 100 kV. The micrograph shows a detail of the cytoplasm of an infect cell harboring an IBDV PVA. Bar corresponds to 200 μm.
(TIF)

S3 Fig Ultrastructural analysis of lysed infected cells. Micrograph from an ultrathin section from QM7 cells infected (3 PFU/cell) with WT IBDV collected at 24 h PI. The image shows three infected cells with intact plasma membranes along with debris released from neighboring lysed cell. Inset shows a higher magnification corresponding to the boxed area containing distinctive IBDV-derived macromolecular assemblages, i.e. paracrystaline virus arrays (PVA), vesicle-containing virions (VCV) and type II VP4 tubules. Inset’s scale bar corresponds to 200 nm. Samples were prepared as described in the Material and Methods section.
(TIF)

S1 Video Real-time cell death analysis of mock-infected cells. Preconfluent QM7 cell monolayers were mock-infected. Following the adsorption period, cultures were incubated in medium supplemented with IncuCyte Cytotox Green reagent that allows the detection of cells exhibiting a damaged PM (green cells). The same monolayer field was monitored every 30 min from 0 to 48 h PI using an IncuCyte ZOOM System apparatus. Captured images were assembled to generate the video recording.
(MP4)

S2 Video Real-time cell death analysis cells infected with the WT virus. Preconfluent QM7 cell monolayers were infected (3 PFU/cell) with the WT virus. Samples were processed and recorded as described in S3 Video.
(MP4)

S3 Video Real-time cell death analysis cells infected with the VP5-KO virus. Preconfluent QM7 cell monolayers were infected (3 PFU/cell) with the VP5-KO virus. Samples were processed and recorded as described in S3 Video.
(MP4)
