# Supplementary figures and images for: Non-Lytic Egression of Infectious Bursal Disease Virus (IBDV) Particles from Infected Cells
Source: PLoS One. 2017 Jan 17;12(1):e0170080. doi: 10.1371/journal.pone.0170080 (PMC5240931; doi:10.1371/journal.pone.0170080)

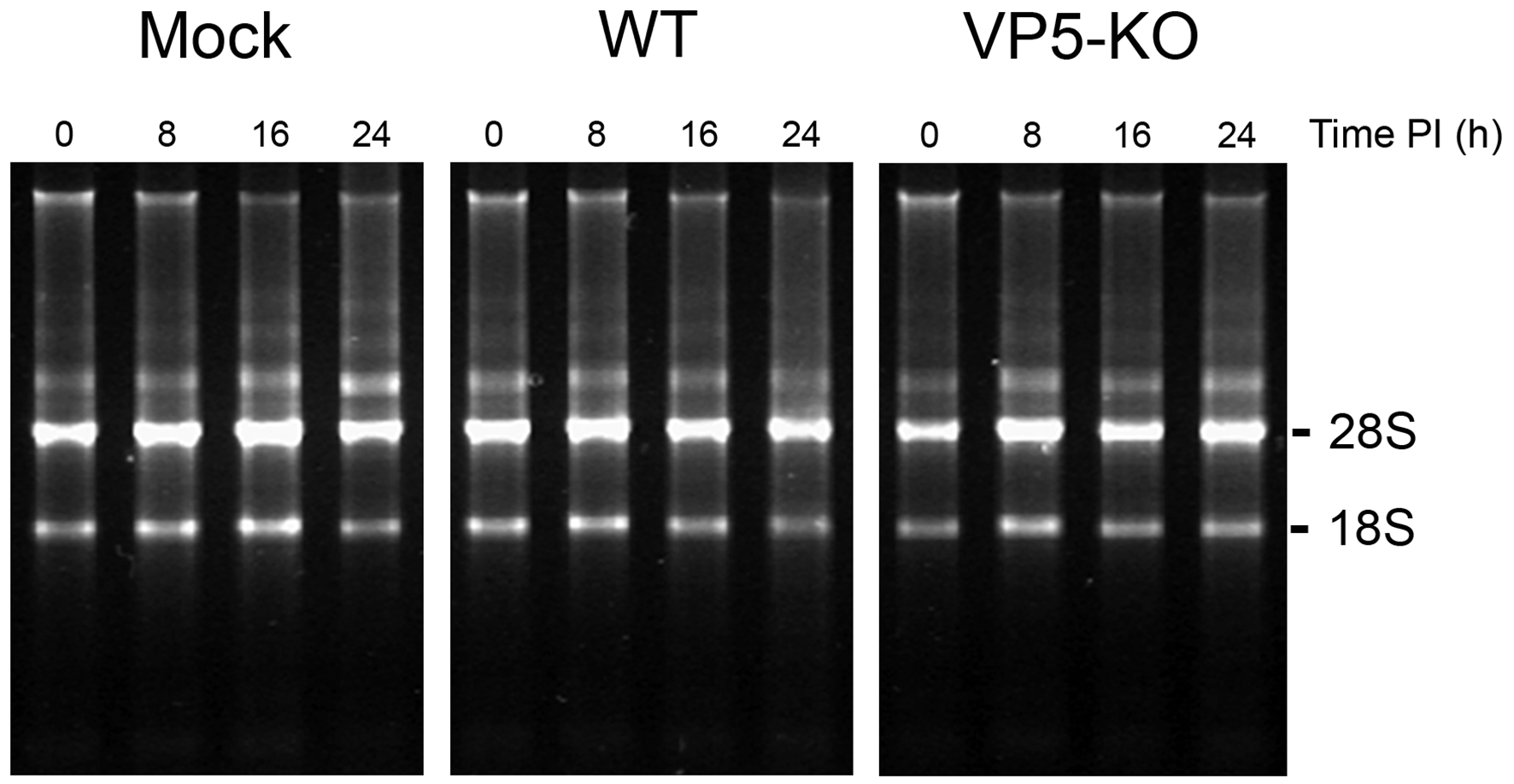

Supplement: S1 Fig — QM7 cell monolayers were mock-infected (Mock) or infected (MOI of 3 PFU/cell) with the WT or the VP5-KO virus. At the indicated times PI, cultures were harvested and processed for the isolation of total RNA using the RNeasy kit (Qiagen). Isolated RNAs were subjected to Tris-borate-EDTA 1% agarose gel electrophoresis. The gel was stained with SYBR Safe (ThermoFisher Scientific). The positions of the 28S and 18S ribosomal RNAs are indicated. (TIF) [file pone.0170080.s001.tif]

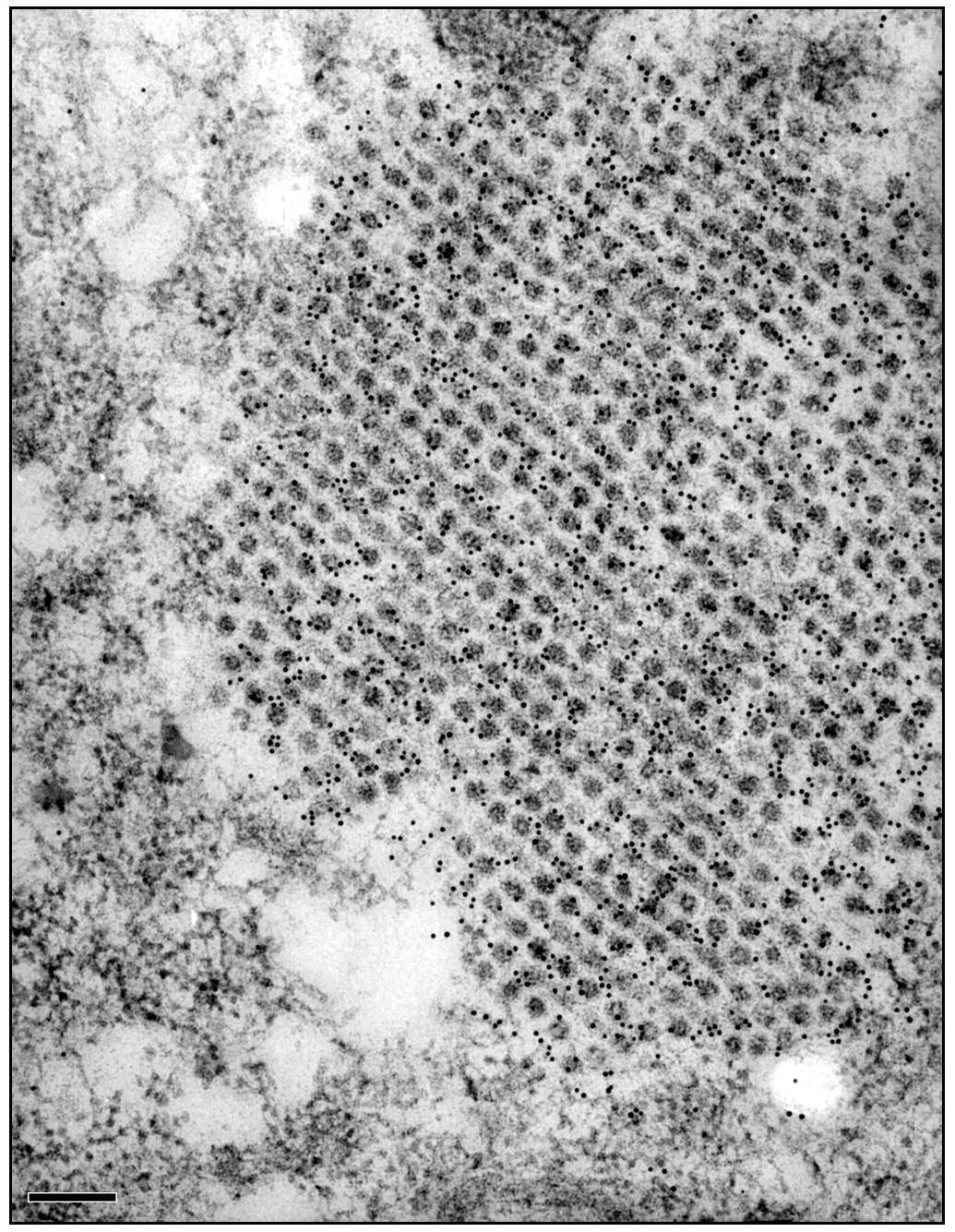

Supplement: S2 Fig — QM7 cells were infected (3 PFU/cell) with WT IBDV. At 16 h PI monolayers were fixed in situ with 4% paraformaldehyde 0.1% glutaraldehyde in PBS at 4°C for 4 h. After fixation, cell pellets were suspended in glycerol and frozen in liquid ethane. Frozen specimens were transferred to a Riechert-Jung AFS freeze-substitution unit (Leika) and maintained at 90°C in a mixture of methanol and 0.5% (W/V) uranyl acetate for 48 h. Thereafter, samples were infiltrated in Lowicryl KYM (EML laboratories) at 30°C. Polymerization was induced with UV light. Ultrathin sections of the samples were immunolabeled with anti-VP2 serum followed by incubation with goat anti-rabbit IgG conjugated to 5-nm colloidal gold. Micrographs were recorded with a Jeol 1200 EXII electron microscope operating at 100 kV. The micrograph shows a detail of the cytoplasm of an infect cell harboring an IBDV PVA. Bar corresponds to 200 μm. (TIF) [file pone.0170080.s002.tif]

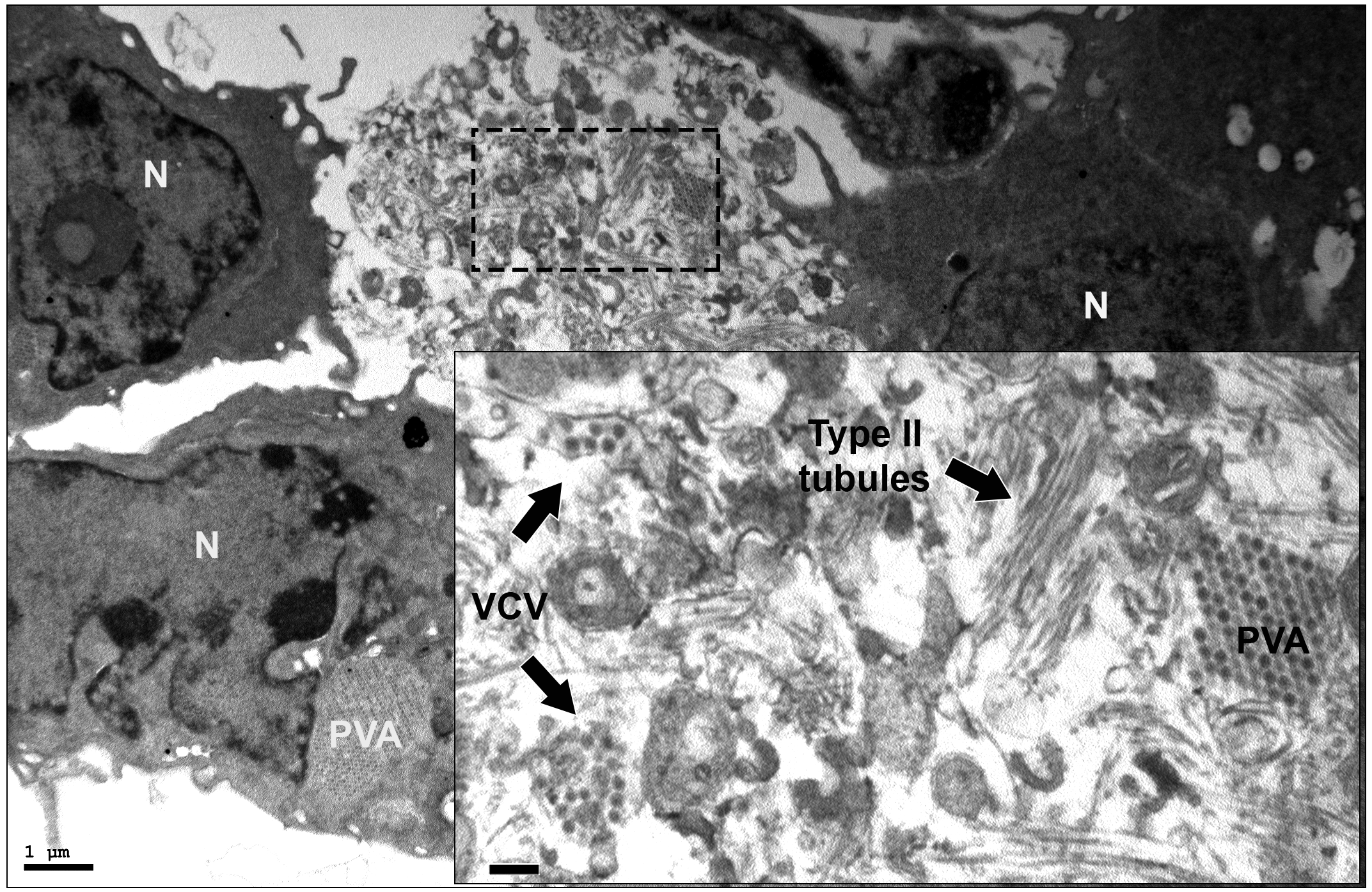

Supplement: S3 Fig — Micrograph from an ultrathin section from QM7 cells infected (3 PFU/cell) with WT IBDV collected at 24 h PI. The image shows three infected cells with intact plasma membranes along with debris released from neighboring lysed cell. Inset shows a higher magnification corresponding to the boxed area containing distinctive IBDV-derived macromolecular assemblages, i.e. paracrystaline virus arrays (PVA), vesicle-containing virions (VCV) and type II VP4 tubules. Inset’s scale bar corresponds to 200 nm. Samples were prepared as described in the Material and Methods section. (TIF) [file pone.0170080.s003.tif]
